# Deciphering the NEK7-NLRP3 inflammasome assembly: from conformational activation to allosteric drug discovery

**DOI:** 10.3389/fimmu.2026.1773422

**Published:** 2026-04-14

**Authors:** Chun-Ling Gu, Dong-Dong Liu, Hong Chen, Xiao-Hong Wei, Hong-Cai Shang

**Affiliations:** 1 Key Laboratory of Chinese Internal Medicine of Ministry of Education and Beijing, Dongzhimen Hospital, Beijing University of Chinese Medicine, Beijing, China; 2 State Key Laboratory of Organ Regeneration and Reconstruction, Institute of Zoology, Chinese Academy of Sciences, Beijing, China; 3 Beijing Institute for Stem Cell and Regenerative Medicine, Beijing, China; 4 Dongfang Hospital, Beijing University of Chinese Medicine, Beijing, China

**Keywords:** inflammasome inhibitors, NEK7, NLRP3 inflammasome, post-translational modification, targeted therapy

## Abstract

The NLRP3 inflammasome, a pivotal component of innate immunity, orchestrates immune defense and inflammatory responses. NEK7, an essential upstream regulator, drives inflammasome assembly through direct interaction with NLRP3. This review systematically summarizes the molecular mechanisms, upstream regulatory networks, and therapeutic targeting of the NEK7-NLRP3 axis. Structurally, NEK7 binds to the leucine-rich repeat (LRR) domain of NLRP3 via its catalytic domain, inducing conformational rearrangement and oligomerization. This structural shift exposes NLRP3’s PYRIN domain (PYD), enabling ASC recruitment through homotypic PYD-PYD interactions and subsequent pro-caspase-1 activation to form the mature inflammasome complex. At the regulatory level, cell cycle-dependent NEK7 availability, post-translational modifications (phosphorylation/ubiquitination/palmitoylation), and numerous upstream signals—including kinases, ubiquitin ligases, ionic fluxes, miRNAs, and pathogens—collectively fine-tune the NEK7-NLRP3 interaction. In terms of therapeutic targeting, natural compounds from traditional Chinese medicine (e.g., oridonin, pristimerin), synthetic inhibitors (e.g., MCC950, entrectinib), and biological agents have been shown to suppress inflammasome activation by disrupting the NEK7-NLRP3 interface or modulating associated regulatory pathways. These advances offer novel therapeutic strategies for NLRP3-driven pathologies including gouty arthritis, ischemia-reperfusion injury, neurodegenerative disorders, and metabolic syndromes.

## Graphical Abstract

**Figure:**
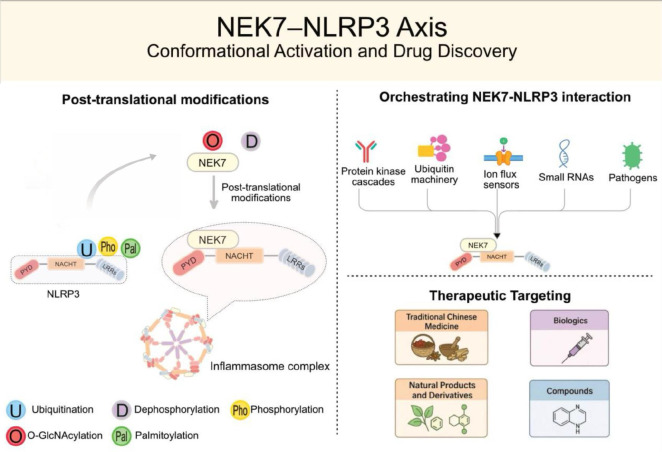

## 1 Introduction

The NOD-like receptor family pyrin domain containing 3 (NLRP3) inflammasome serves as a pivotal regulator of host defense, orchestrating immune responses against infection, metabolic disorders, and immune homeostasis by mediating the maturation and release of pro-inflammatory cytokines interleukin- 1β (IL-1β) and interleukin- 18 (IL-18), as well as executing pyroptosis (1). Structurally, the NLRP3 inflammasome assembly requires three core components: the pattern recognition receptor NOD (nucleotide-binding oligomerization domain), LRR (leucine-rich repeat) and PYD (pyrin domain)-containing protein 3, the adaptor protein ASC (apoptosis-associated speck-like protein containing a caspase recruitment domain, and the effector protein pro-caspase-1 (2).

NLRP3 is a modular protein composed of three functional domains: an N-terminal PYD domain that mediates protein-protein interactions, a central nucleotide-binding and oligomerization domain (NACHT) that governs conformational dynamics, and a C-terminal LRR domain responsible for ligand sensing and maintaining autoinhibitory constraints in the resting state (3). In its inactive conformation, intramolecular interactions between the LRR and NACHT domains maintain NLRP3 in a closed, cage-like structure that restricts oligomerization, with both NLRP3 and ASC diffusely localized within the cytoplasm.

Upon activation, the LRR domain undergoes conformational rearrangement that relieving autoinhibitory constraints and exposes the NACHT domain, thereby promoting NLRP3 self-oligomerization. This oligomerized NLRP3 scaffold subsequently facilitates ASC recruitment through homotypic PYD-PYD interactions. Concurrently, ASC’s C-terminal CARD domain engages the CARD domain of pro-caspase-1, driving the formation of a supramolecular inflammasome complex characterized by perinuclear speck-like structures (~1 µm in diameter) (4). Within this complex, pro-caspase-1 undergoes proximity-induced autoactivation, exerting its biphasic effector function: cleaving gasdermin D (GSDMD) to generate pore-forming fragments that induce pyroptotic cell lysis, and processing pro-IL-1β/pro-IL-18 into their bioactive mature forms to initiate a robust inflammatory cascade (5).

**Figure 1:**
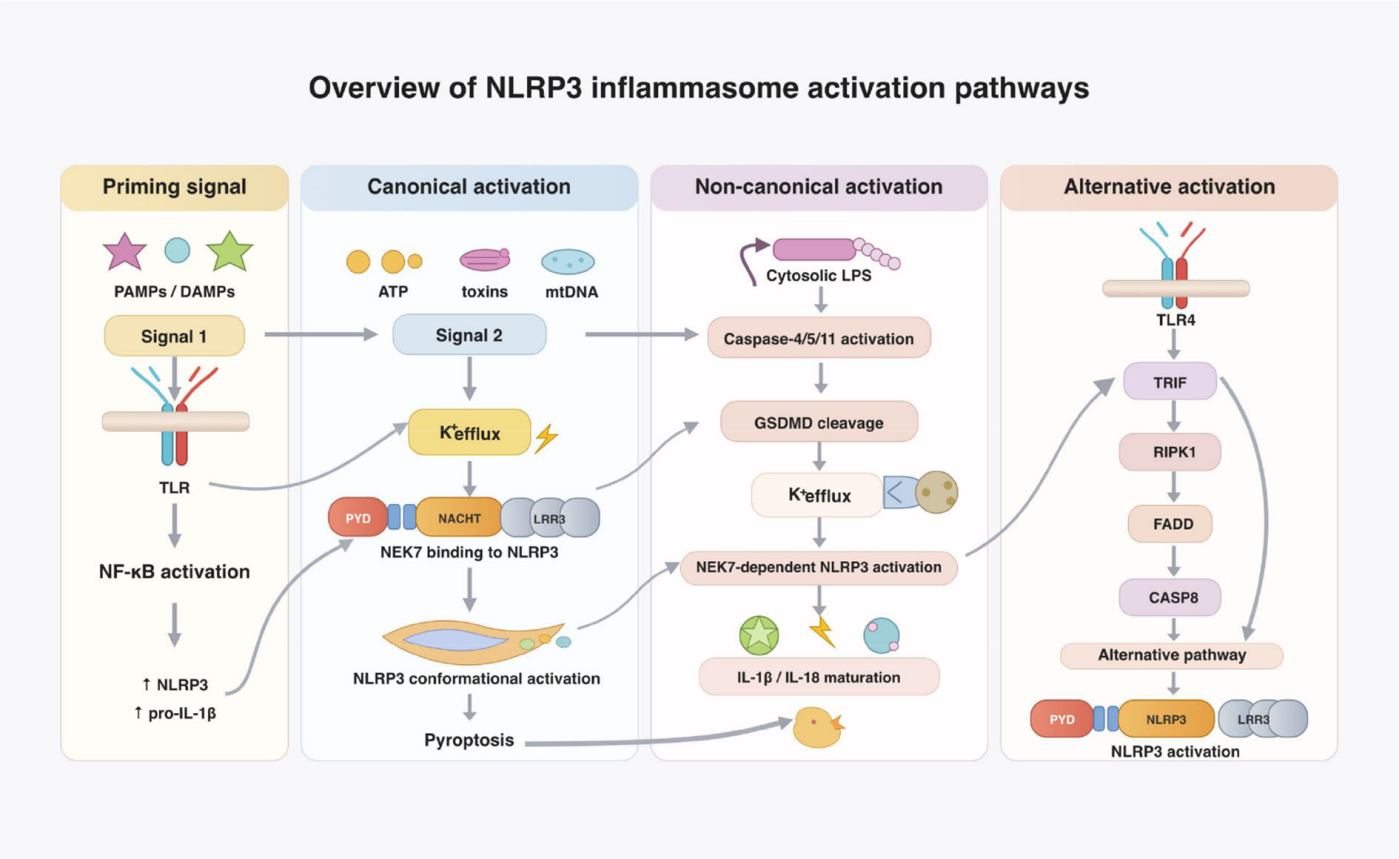
Overview of NLRP3 inflammasome activation pathways. NLRP3 inflammasome activation occurs through canonical, non-canonical, and alternative pathways. During the priming step, pathogen-associated molecular patterns (PAMPs) or danger-associated molecular patterns (DAMPs) activate Toll-like receptor (TLR) signaling and NF-κB, inducing transcriptional upregulation of NLRP3 and pro-IL-1β. In the canonical pathway, diverse stimuli such as ATP, toxins, and mitochondrial damage trigger potassium efflux, allowing NEK7 to bind the LRR domain of NLRP3 and promote inflammasome assembly. In the non-canonical pathway, cytosolic lipopolysaccharide (LPS) activates caspase-4/5 in humans or caspase-11 in mice, leading to gasdermin D cleavage and secondary potassium efflux that subsequently activates NLRP3. The alternative pathway involves TLR4-TRIF signaling and caspase-8 activation, resulting in NLRP3 inflammasome activation without classical pyroptosis.

The NLRP3 inflammasome can be activated through three major pathways: canonical, non-canonical, and alternative. The NLRP3 inflammasome can be activated through three major pathways: canonical, non-canonical, and alternative, each triggered by distinct upstream stimuli and signaling cascades (**Figure 1**). The canonical pathway operates through a two-step process. First, pathogen-associated molecular patterns (PAMPs) such as lipopolysaccharide (LPS) or inflammatory stimuli acting through Toll-like receptor signaling, trigger nuclear factor kappa-B (NF-κB) activation and thereby induce the expression of NLRP3 and pro-IL-1β, which licenses subsequent inflammasome assembly and cytokine maturation{Kelley, 2019 #7166}. Subsequently, a second set of stimuli, including extracellular ATP, bacterial toxins, or mitochondrial DNA, triggers NLRP3 oligomerization and PYD–PYD-mediated ASC recruitment, followed by CARD–CARD-mediated caspase-1 activation. Activated caspase-1 cleaves GSDMD to form membrane pores, facilitating the release of IL-1β/IL-18 and triggering pyroptosis (6, 7).

In contrast, the non-canonical pathway is initiated when cytosolic LPS derived from Gram-negative bacteria directly activates caspase-4/5 in humans or caspase-11 in mice. Activates caspase-4/5/11cleaves GSDMD, causing membrane pore formation, pyroptosis, and potassium efflux. The resulting decrease in intracellular K^+^ acts as a key secondary signal that enables NEK7 binding, NLRP3 oligomerization, ASC recruitment, and subsequent caspase-1 activation. The alternative pathway depends on TLR4– TIR-domain-containing adapter-inducing interferon-β (TRIF)– receptor-interacting serine/threonine-protein kinase 1 (RIPK1)– Fas-associated death domain protein (FADD)– caspase-8 (CASP8) signaling axis to activate NLRP3 (8). In Addition to these pathways, NLRP3 inflammasome activation is regulated by multiple cellular stress signals, including K^+^/Cl^-^ efflux, reactive oxygen species (ROS), Ca^2+^ signaling, mitochondrial dysfunction, and lysosomal rupture (9–12).

Among the regulatory proteins involved in this process, NIMA-related kinase 7 (NEK7) has emerged as an indispensable structural licensing factor for NLRP3 inflammasome assembly (13–15). Originally characterized for its role in mitotic entry and spindle organization, NEK7 directly binds to the leucine-rich repeat (LRR) domain of NLRP3 through its catalytic domain, promoting the transition of NLRP3 from an autoinhibited cage-like conformation to an open, oligomerization-competent state. This conformational rearrangement exposes the PYD domain, thereby enabling ASC recruitment and subsequent inflammasome assembly.

Importantly, the role of NEK7 varies across the different NLRP3 activation pathways. In the canonical pathway, NEK7 functions as a critical downstream effector of potassium efflux and serves as a licensing factor for NLRP3 oligomerization. In the non-canonical pathway, caspase-4/5/11-mediated GSDMD cleavage induces secondary potassium efflux, which subsequently creates a permissive environment for NEK7-dependent NLRP3 activation. In the alternative pathway, NEK7 is also implicated in inflammasome activation, although its precise structural contribution remains less clearly defined. These findings position NEK7 as a convergent molecular checkpoint linking diverse upstream danger signals to the conformational activation of NLRP3.

## 2 Core molecular mechanisms underlying the NEK7-NLRP3 interaction

### 2.1 Structural basis of NEK7-NLRP3 interaction

NEK7 serves as a structural driver essential for NLRP3 inflammasome assembly. Under cellular stress, ATP hydrolysis triggers NLRP3 conformational changes, exposing its LRR domain to specifically bind the catalytic domain of NEK7, thereby forming the functional NEK7-NLRP3 complex and inducing oligomerization. This interaction further promotes NLRP3 oligomerization, exposing its PYD domain to recruit ASC and procaspase-1, thus completing inflammasome assembly (16). Genetic deletion of the LRR domain disrupts NLRP3 self-association, oligomerization, and its interaction with NEK7 (17). Notably, a single residue (arginine 121, R121) within the catalytic domain of NEK7 is indispensable for the specificity of this interaction, allowing it to discriminate precisely between NEK7 and its close homolog NEK6 during inflammasome activation (18). Importantly, the structural interaction between NEK and NLRP3 is closely coupled with the spatial redistribution of NLRP3 with the cell. In resting cells, NLRP3 is mainly localized in the cytosol and membrane compartments. Upon activation, NLRP3 undergoes dynamic trafficking through the dispersed trans-Golgi network and mitochondria-associated membranes before accumulating at the microtubule-organizing center (MTOC) (19–21). This spatial organization provides a platform for NEK7 recruitment and facilitates the assembly of the NLRP3 inflammasome.

Structural studies reveal that NLRP3 activation requires domain rotation and assembly within NEK7-bound NLRP3 monomers. This domain rotation represents an “uphill” energy state, in which the active conformation of NEK7-bound NLRP3 monomers is inherently unstable. However, within a disk-like oligomeric complex possessing C10 symmetry, the active conformation of NEK7-NLRP3 becomes stabilized. This stability is maintained by inter-subunit interactions termed the “FISNA loop–alligator clip” mechanism, wherein the FISNA loop 1 of one subunit interacts with an “alligator clip” structure in a neighboring subunit—composed of an NBD helix-loop-strand motif (residues 351–373) and a WHD β-hairpin loop (residues 501–521) (22).

### 2.2 Post-translational modifications regulating the NEK7-NLRP3 interaction

Post-translational modifications (PTMs) act as dynamic molecular switches that regulate the licensing, strength, and spatial organization of the NEK7-NLRP3 interactions. Rather than functioning as isolated regulatory events, PTMs on NEK7 and NLRP3 cooperatively modulate inflammasome activation by altering protein conformation, interaction affinity, and subcellular localization. Accumulating evidence indicates that phosphorylation, palmitoylation, ubiquitination, O-GlcNAcylation, and redox-related modifications operate in a context dependent and coordinated manner to fine-tune NEK7-dependent NLRP3 inflammasome assembly.

#### 2.2.1 PTMs of NEK7

As a essential licensing factor for NLRP3 inflammasome activation, NEK7 undergoes precise PTMs that regulate both its availability and interaction competence. O-GlcNAcylation of NEK7 at serine residues S204 and S260, mediated by O-GlcNAc transferase (OGT), inhibits phosphorylation at S260 and consequently enhances the binding affinity of NEK7 to NLRP3 (23, 24). In parallel, redox regulation play a critical role: deglutathionylation at cysteine residue C253 significantly increases NEK7 activity toward NLRP3, thereby promoting inflammasome activation (25). In addition to site-specific modification, metabolic-state-dependent regulation of NEK7 further influences inflammasome signaling. Sirtuin 5 (Sirt5) suppresses NEK7 expression by promoting desuccinylation at lysine 81 (K81), leading to reduced levels of NLRP3, ASC, and Caspase-1 in a mouse model of middle cerebral artery occlusion (26). Moreover, epitranscriptomic regulation indirectly modulates NEK7 activity. In periodontitis, methyltransferase-like 3 (METTL3) facilitates N6-methyladenosine (m6A) modification-mediated degradation of tumor necrosis factor alpha-induced protein 3 (TNFAIP3) transcripts, thereby reducing the expression of this anti-inflammatory E3 ligase. Because TNFAIP3 ubiquitinates NEK7 to suppress inflammasome activation, METTL3-driven TNFAIP depletion ultimately promotes NEK7-NLRP3 inflammasome activation (27). Together, these findings indicate that PTMs on NEK7 primarily regulate interaction permissiveness and functional availability, thereby licensing NLRP3 inflammasome activation in response to inflammatory and metabolic cues.

#### 2.2.2 PTMs of NLRP3

NLRP3 PTMs (phosphorylation, palmitoylation, and ubiquitination) critically regulate its subcellular localization and interaction strength with NEK7, thereby serving as central nodes in the control of inflammasome activation.

Phosphorylation is a key mechanism regulating the NEK7-NLRP3 interaction. The phosphorylation state of serine 803 (S803), located at the interface between NLRP3 and NEK7, directly determines NEK7 recruitment. During the priming phase, phosphorylation at S803 promotes NEK7 binding, while dephosphorylation during the activation phase relieves this inhibition. Notably, phosphomimetic mutations at the S803 site have been shown to significantly impair NEK7 recruitment and suppress NLRP3 inflammasome activity (28). Moreover, upon NLRP3 activation, potassium efflux and the downstream activation of JNK1 via GSDMD lead to rapid phosphorylation of NEK7 at threonine residues 190 and 191. This modification strengthens the NEK7-NLRP3 interaction and further facilitates inflammasome assembly and activation (29).

Palmitoylation also plays a vital role in modulating NLRP3’s subcellular localization and interaction potential. Palmitoylation of NLRP3 at cysteine 419 (C419), catalyzed by palmitoyltransferase zinc finger DHHC-type palmitoyltransferase 17 (ZDHHC17), enhances NEK7 recruitment and promotes their interaction, thereby triggering NLRP3 inflammasome activation (30). Interestingly, additional palmitoylation at cysteine residues 958 and 130 (C958 and C130) occurs during both the priming and activation phases. This modification targets NLRP3 to specific membrane domains and ultimately localizes it to the MTOC. There, it acts in coordination with large tumor suppressor kinases 1 and 2 (LATS1/2), which are pre-localized to the MTOC and mediate S265 phosphorylation during priming, synergistically promoting the NEK7-NLRP3 interaction and inflammasome activation (31).

Ubiquitination, in contrast, serves as a negative regulatory mechanism of the NEK7-NLRP3 interaction. Tripartite motif-containing protein 65 (TRIM65) has been shown to bind the NACHT domain of NLRP3 and mediate K48- and K63-linked ubiquitination, which interferes with NEK7 binding and significantly suppresses NLRP3 inflammasome activation (32).

**Figure 2:**
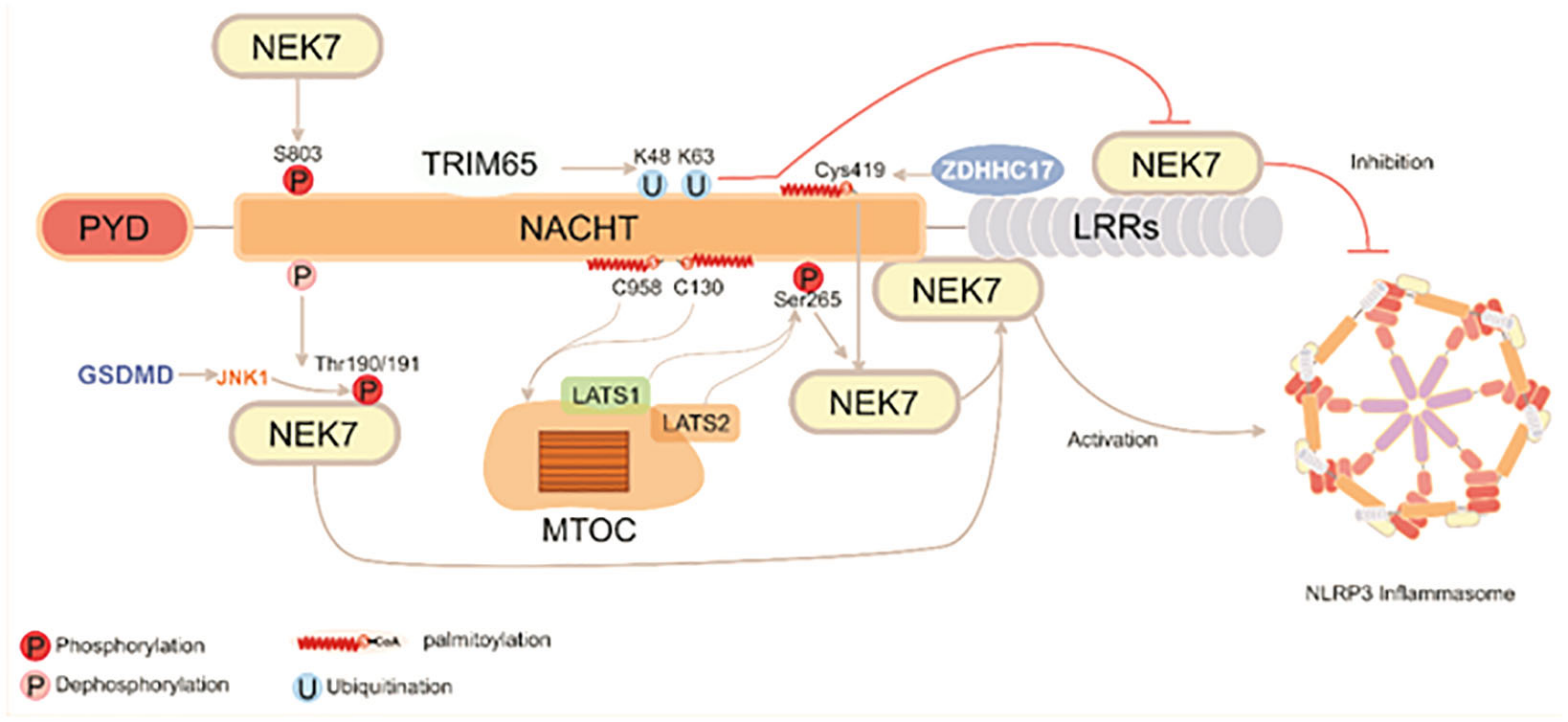
Schematic illustration of the regulatory mechanisms by which NEK7 mediates NLRP3 inflammasome activation. NEK7 interacts with multiple structural domains of NLRP3 and participates in its regulation through phosphorylation, palmitoylation, and ubiquitination at various sites. TRIM65, ZDHHC17, and the LATS1/2 complex coordinately modulate the assembly and activation of the NLRP3 inflammasome, ultimately triggering GSDMD-mediated pyroptosis.

Collectively, these findings (**Figure 2**) highlight that PTMs of both NEK7 (including O-GlcNAcylation and deglutathionylation) and NLRP3 (such as phosphorylation, palmitoylation, and ubiquitination) orchestrate the strength and localization of their interaction, acting as pivotal regulatory checkpoints in NLRP3 inflammasome activation.

## 3 NEK7–NLRP3 axis: upstream regulatory network

### 3.1 Protein kinases and signaling pathways

Protein kinase–mediated signaling pathways constitute a major regulatory layer controlling the NEK7–NLRP3 axis. Although phosphate phosphorylation and ubiquitination represent classical post-translational modifications (PTMs), many upstream signaling pathways regulate the NEK7–NLRP3 interaction through kinase activation or E3 ubiquitin ligase–mediated signaling cascades. In this section, we therefore focus on the upstream signaling context that modulates the NEK7–NLRP3 interaction, whereas the detailed molecular mechanisms of PTMs are discussed separately in Section 2.2.

Current evidence suggests that these pathways regulate inflammasome activation through three principal mechanisms: (i) modulation of NLRP3 conformational activation, (ii) stabilization of the NEK7–NLRP3 interaction interface, and (iii) amplification of downstream inflammatory signaling cascades.

Reactive oxygen species(ROS), which function as central mediators of NLRP3 inflammasome activation, not only participate in inflammatory signal transduction but also directly influence the interaction between NEK7 and NLRP3. ROS-dependent redox signaling contributes to the conformational stability of the NEK7-NLRP3 complex, thereby facilitating inflammasome assembly (33).

In addition, scaffold proteins and kinase signaling modules play key roles in organizing inflammasome activation. For instance, receptor for activated C kinase 1(RACK1) acts as a molecular scaffold that simultaneously interacts with NEK7 and NLRP3,promoting the transition of NLRP3 from an autoinhibited conformation to an active oligomerization-competent state (34).

Furthermore, kinase-driven phosphorylation events can enhance the interaction between NEK7 and NLRP3. These phosphorylation-dependent regulatory mechanisms represent important signaling inputs that influence the stability and activation of the NEK7–NLRP3 complex. For example, p21-activated kinases 1 and 2 (Pak1/2) regulate the NEK7-NLRP3 axis through phosphorylation-dependent mechanisms that promote inflammasome activation and the maturation of pro-inflammatory cytokines such as IL-1β (35).

Upstream stress-responsive kinases also contribute to inflammasome regulation during pathogenic infection. For instance, during *Listeria monocytogenes* infection, mammalian sterile 20-like kinases 1 and 2 (Mst1/2) and anaplastic lymphoma kinase (ALK) amplify the NEK7-NLRP3 interaction through activation of the JNK signaling pathway. JNK-mediated phosphorylation stabilizes the NEK7-NLRP3 complex and facilitates robust pro-inflammatory cytokine release, thereby contributing to host defense against intracellular pathogens (36).

In Addition to these activating mechanisms, several kinase-associated pathways exert inhibitory effects on inflammasome activation. further illustrating that kinase signaling networks function as upstream modulators of the NEK7–NLRP3 axis. RIPK1 interacts with IκBα to regulate NEK7-NLRP3 binding in macrophages, whereas deficiency of RIPK1 or apoptosis signal-regulating kinase 1 (ASK1) has been reported to attenuate inflammatory responses by reducing HMGB1 release (37). Moreover, in retinal endothelial cells, exchange protein directly activated by cAMP 1 (Epac1) and protein kinase A (PKA) suppress NEK7-NLRP3 signaling through cAMP-dependent pathway (38).

### 3.2 Ubiquitination-related molecules

Ubiquitination-dependent regulation represents another important mechanism controlling the NEK7–NLRP3 inflammasome axis. Current evidence suggests that ubiquitination-related molecules regulate inflammasome activation through several coordinated mechanisms, including (i) ubiquitin-mediated degradation of inflammasome components, (ii) interference with the NEK7–NLRP3 interaction interface, and (iii) transcriptional or signaling modulation of inflammasome-associated proteins.

Among these regulators, A20 (TNFAIP3) functions as a key negative regulator of inflammasome activation. A20 suppresses NEK7 activity through multiple mechanisms. First, it promotes proteasomal degradation of NEK7 by mediating its ubiquitination at lysine residues K189 and K293. Second, A20 disrupts the formation of the NEK7-NLRP3 complex through the coordinated action of its OTU domain and ZnF4/ZnF7 motifs. Third, A20 downregulates basal NEK7 expression, ultimately limiting NLRP3 inflammasome activation (39).

In contrast, deubiquitination-related signaling may exert distinct regulatory effects on the NEK7-NLRP3 axis. For example, deubiquitinated polo-like kinase(PLK4) exhibits enhanced interaction and phosphorylation with NEK7, which subsequently reduces NEK7 binding affinity for NLRP3 and suppresses inflammasome activation (40).

In addition, ubiquitination-associated transcriptional regulation also contributes to inflammasome control. Ubiquitin-specific peptidase 22 (USP22) regulates NLRP3 inflammasome activity through deubiquitination-dependent activation of the transcription factor PU.1 (PU box binding protein 1). By activating PU.1, USP22 indirectly modulates the expression of inflammasome components and inhibits NLRP3 inflammasome assembly. Consistently, overexpressing USP22 leads to reduced protein levels of NLRP3, NEK7, Caspase-1, and ASC (41).

At the organelle level, mitochondrial E3 ubiquitin ligase also participate in inflammasome regulation. For instance, the mitochondria-localized ligase membrane-associated RING-CH-type finger 5 (MARCH5) mediates the ubiquitination of NLRP3, a modification that facilitates NEK7-NLRP3 complex formation and promotes NLRP3 oligomerization (42). In contrast, the HECT domain E3 ubiquitin protein ligase 3 (HECTD3) negatively regulates inflammasome activation through a mechanism independent of its E3 ligase activity. The DOC domain of HECTD3 directly interacts with the NACHT/LRR domains of NLRP3, thereby blocking the NEK7-NLRP3 interface and inhibiting inflammasome assembly (43).

Collectively, ubiquitination-related factors—including A20, PLK4, USP22, MARCH5, and HECTD3—finely tune the NEK7-NLRP3 interaction through diverse mechanisms such as targeted degradation, interaction blockade, transcriptional modulation, and subcellular localization control. These findings highlight the central role of ubiquitination-dependent signaling in maintaining the balance of inflammasome activation.

### 3.3 Ion channels and ionic flux signaling

Ion channel–mediated ionic flux represents one of the most fundamental triggers of NLRP3 inflammasome activation and plays a critical role in regulating the NEK7–NLRP3 interaction. Current evidence indicates that ion channels modulate inflammasome activation primarily through potassium (K^+^) efflux–dependent signaling and downstream ionic regulatory pathways that facilitate the assembly of the NEK7–NLRP3 complex.

Among these regulators, the P2X purinoceptor 7 (P2X7) receptor functions as a key upstream sensor that promotes NLRP3 inflammasome activation by mediating potassium (K^+^) efflux. Activation of P2X7 induces rapid K^+^ depletion in the cytoplasm, which facilitates the interaction between NEK7 and NLRP3 and subsequently drives inflammasome assembly and activation (44).

In addition to potassium signaling, intracellular chloride (Cl^−^) channels also participate in inflammasome regulation. These channels act downstream of the “K^+^ efflux–mitochondrial reactive oxygen species (mtROS)” axis and enhance the binding affinity between NEK7 and NLRP3, thereby facilitating NLRP3 inflammasome activation (45). However, emerging evidence suggests that NEK7 functions as a specific K^+^ sensor whose activity is strictly dependent on K^+^ efflux. Under conditions of K^+^ depletion, NEK7 can bind to NLRP3 and initiate inflammasome assembly, whereas Cl^−^ efflux alone is insufficient to induce this interaction (46).

This icon flux-dependent regulatory mechanism is particularly evident in macrophages carrying NLRP3 gain-of-function mutations associated with cryopyrin-associated periodic syndrome (CAPS), such as R258W mutation. In these cells, NEK7 is essential for caspase-1 activation. Upon stimulation, macrophages form high-molecular-weight NEK7-NLRP3 complexes accompanied by ASC oligomerization and the formation of ASC specks, whereas these processes are abolished in the absence of NEK7 (47).

Further studies have demonstrated that NEK7 mediates a potassium efflux-responsive signaling cascade that including NEK7-NLRP3 complex formation, pro-caspase-1 recruitment, caspase-1 activation, and pyroptotic cell death. This pathway ultimately triggers NLRP3 inflammasome activation and contributes to inflammatory responses such as neuroinflammation following traumatic brain injury (48).

Collectively, these findings highlight that ion channel–mediated ionic flux, particularly potassium efflux, serves as a critical upstream signal that licenses NEK7–NLRP3 inflammasome assembly and links cellular stress signals to inflammatory responses.

### 3.4 MicroRNAs and epigenetic regulation

MicroRNA-mediated post-transcriptional regulation represents another important mechanism modulating the NEK7–NLRP3 inflammasome axis. Emerging evidence indicates that several microRNAs regulate inflammasome activation by directly targeting NEK7 mRNA and suppressing its expression, thereby limiting NEK7-dependent inflammasome assembly.

For example, miR-340 directly targets NEK7 and suppresses NLRP3 inflammasome signaling (49). Similarly, miR-214-3p, a class of endogenous small non-coding RNA, binds to the 3′ untranslated region (3′UTR) of NEK7 mRNA, thereby inhibiting NEK7 expression and suppressing inflammasome activation (50). In metabolic disease models, miR-23a-3p has also been reported to attenuate NLRP3-induced pyroptosis by targeting NEK7, thereby alleviating hepatic and renal injury in type 2 diabetes rats (51).

In addition to direct regulation of NEK7, certain microRNAs can influence the NEK7–NLRP3 axis through broader signaling networks. For instance, miR-181a-5p suppresses LPS-induced pyroptosis in HK-2 cells by downregulating NEK7 expression (52). Moreover, miR-181a-5p can also target USP15 and regulate the USP15/RelA/NEK7/NLRP3 signaling pathway, thereby reducing inflammation and pyroptosis in microglial cells and alleviates disease severity in experimental autoimmune encephalomyelitis (EAE) models (53).

Collectively, these findings suggest that microRNA-dependent epigenetic regulation represents an important regulatory layer controlling NEK7 expression and inflammasome activation under pathological conditions.

### 3.5 Regulation by bacterial and viral pathogens

Bacterial and viral pathogens represent important upstream that modulate the NEK7-NLRP3 inflammasome pathway and shape host inflammatory response. Increasing evidence indicates that pathogens regulate this axis through distinct molecular strategies, including ion flux–dependent activation, scaffold-mediated complex assembly, and competitive inhibition of inflammasome components.

During bacterial infection, several pathogens promote inflammasome activation by facilitating the interaction between NEK7 and NLRP3. For example, *Pasteurella multocida* serogroup A enhances NEK7-NLRP3 binding through potassium (K^+^) efflux-dependent mechanisms (54). Similarly, *Staphylococcus aureus* activates a K^+^ efflux/Syk/JNK signaling cascade that promotes NEK7-NLRP3 complex formation and the release of pro-inflammatory cytokines (55). In addition, *Streptococcus suis* induces the formation of a RACK1-NEK7-NLRP3 complex, thereby activating the NLRP3 inflammasome and amplifying inflammatory injury through gasdermin D (GSDMD)-mediated pyroptosis (56). In this context, NEK7 contributes to pyroptotic progression by stabilizing the inflammasome complex structure.

In contrast, viral pathogens exhibit bidirectional regulation of the NEK7-NLRP3 axis. Certain viral proteins inhibit inflammasome activation to facilitate immune evasion. For instance, the PB1-F2 protein encoded by the PB1 gene segment of Influenza A virus (IAV PB1-F2) suppresses NEK7 activation and thereby blocks NLRP3 inflammasome assembly (57). Similarly, the rabies virus M protein competes with NEK7 for binding to NLRP3 via its serine 158 residue, disrupting the NEK7-NLRP3 interface and suppressing ASC oligomerization (58, 59). Conversely, other viral factors can enhance inflammasome activation. the viroporin protein of SARS-CoV-2 cooperates with K^+^ efflux to strongly activate NEK7 and the NLRP3 inflammasome, thereby contributing to excessive inflammatory responses such as cytokine storms (60).

Collectively, these findings suggest that bacterial pathogens predominantly activate the NEK7-NLRP3 pathway to enhance immune clearance, whereas viruses adopt dual regulatory strategies—either suppressing inflammasome activation for immune evasion or inducing excessive activation to drive inflammatory pathology. These mechanisms highlight the central role of the NEK7-NLRP3 axis in pathogen–host interactions.

### 3.6 Other regulatory factors

In addition to the regulatory mechanisms described above, various microenvironmental signals and organelle-associated factors further modulate the NEK7–NLRP3 axis through spatial localization, metabolic signaling, and inter-organelle communication.4

Local inflammatory microenvironment signals can influence inflammasome activation by regulating NEK7 transcription or NLRP3 recruitment. For example, amyloid-β (Aβ), through activation of the P2X7 receptor on BV-2 microglial cells, triggers NLRP3 inflammasome activation and induces histone H4K12 lactylation, thereby enhancing NEK7 transcription. Conversely, inhibition of histone lactylation reduces NEK7 transcriptional activity and mRNA expression (61).

Organelle-associated signaling pathways also participate in inflammasome regulation. In mitochondria-related signaling, cytochrome c, anchored to the inner mitochondrial membrane via cardiolipin interacts with the LRR domain of NLRP3, recruiting it to mitochondria and promoting inflammasome assembly. Interestingly, this recruitment simultaneously attenuates NLRP3 interactions with cardiolipin and NEK7, suggesting a dynamic equilibrium in inflammasome regulation (62). In the cytosol, calcitonin gene-related peptide (CGRP) directly bind to NLRP3 and disrupts the NEK7-NLRP3 complex (63).

Metabolic stress-associated factors further modulate the NEK7-NLRP3 signaling axis. For instance, S100A9 activates the TLR2/ATF4 signaling pathway, promoting ATF4 nuclear translocation in macrophages and enhancing NEK7-NLRP3 inflammasome activation, thereby aggravating hepatic ischemia-reperfusion (I/R) injury in non-alcoholic fatty liver disease (64). In contrast, Foxo1 regulates the Hedgehog/Gli1/Snail signaling pathway to modulate RIPK3 activity and suppress NEK7/NLRP3-mediated inflammation during hepatic I/R injury (65).

Lipid mediators and mitochondrial transport proteins also contribute to inflammasome regulation. Various isoforms of platelet-activating factor (PAF) and PAF-like lipids can activate the NLRP3 inflammasome via NEK7, leading to the release of pro-inflammatory cytokines such as IL-1β and IL-18 (66). In contrast, the mitochondrial phosphate transporter SLC25A3 promotes NLRP3 ubiquitination through direct interaction, thereby disrupting the NEK7-NLRP3 complex and negatively regulating inflammasome activation (67).

Finally, cell cycle regulation also influences the NEK7-NLRP3 interaction. NEK7 plays dual roles in mitosis and inflammasome activation (68). Due to its limited cellular abundance, a mutually exclusive regulatory mechanism exists in which NEK7 is preferentially allocated for mitotic functions during cell division, thereby suppressing NLRP3 activation. Conversely, during inflammatory responses, cell division is suppressed to prioritize inflammasome activation. This regulatory strategy ensures coordination between cell cycle progression and inflammation signaling, preventing excessive inflammatory damage (69).

**Figure 3:**
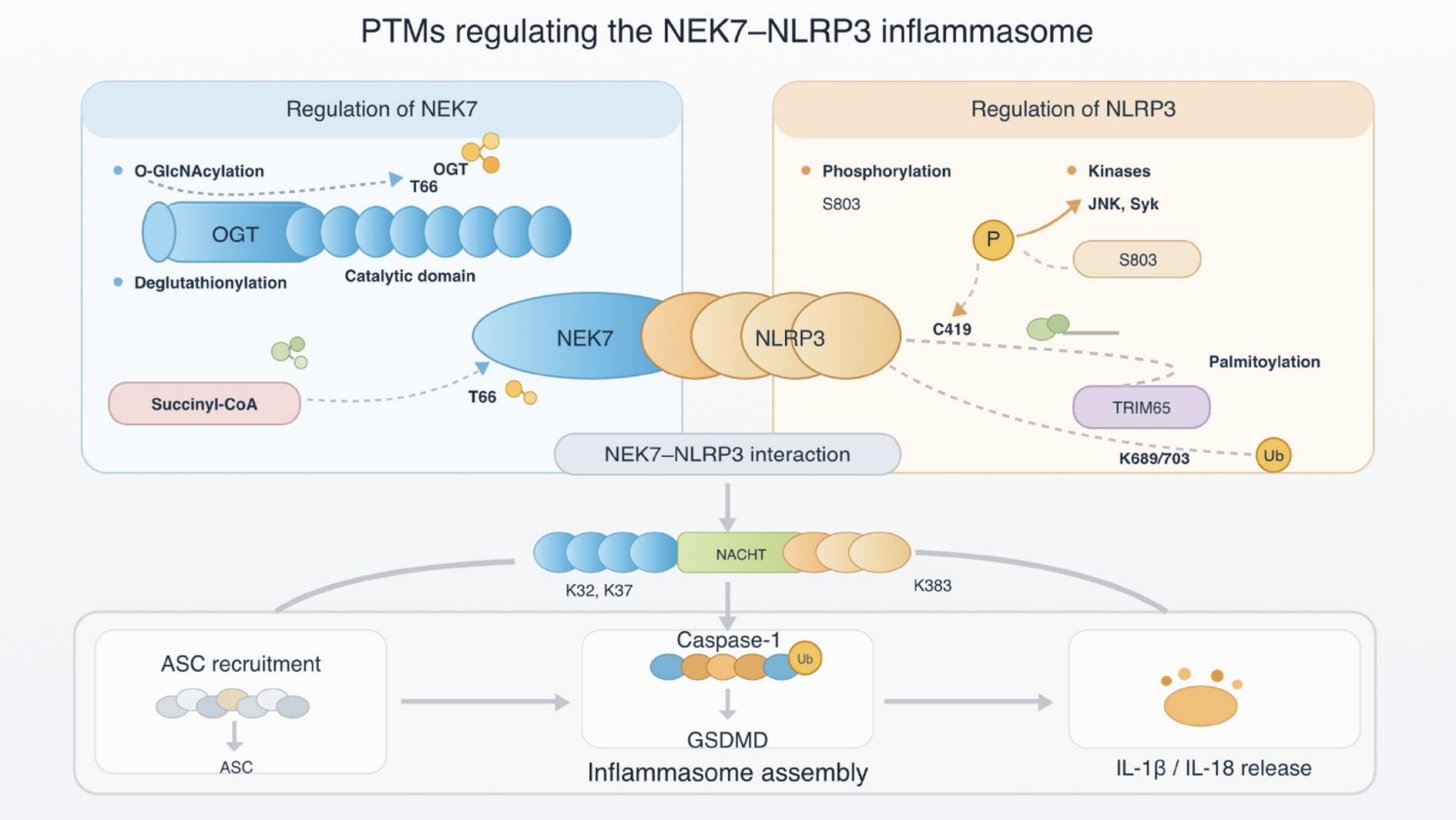
Post-translational modification landscape regulating the NEK7–NLRP3 inflammasome. Post-translational modifications (PTMs) of NEK7 and NLRP3 act as key regulatory switches controlling inflammasome assembly. O-GlcNAcylation, deglutathionylation and metabolic modifications modulate NEK7 activity and its licensing capacity for NLRP3 activation. In parallel, phosphorylation, palmitoylation and ubiquitination dynamically regulate NLRP3 conformational activation and its interaction with NEK7. These PTM-dependent mechanisms converge on the NEK7–NLRP3 interface, which functions as a molecular licensing checkpoint for inflammasome assembly. Upon activation, NEK7-bound NLRP3 oligomers recruit ASC and activate caspase-1, leading to gasdermin D (GSDMD) cleavage, pyroptotic cell death and the release of IL-1β and IL-18.

Collectively, these microenvironmental, metabolic, and organelle-associated regulatory mechanisms further expand the complexity of NEK7-mediated NLRP3 inflammasome activation. As summarized in **Table 1** and illustrated in **Figure 3**, a wide range of molecular and environmental signals converge on NEK7 to fine-tune inflammasome activity.

**Table 1:** Classification of factors regulating the interaction between NEK7 and the NLRP3 inflammasome.

Regulatory category	Key molecules	Brief mechanistic description
Protein kinase–related molecules	NEK7, RACK1, Pak1/2, Mst1/2, ALK, etc.	Promote NEK7-NLRP3 binding or phosphorylation, activating the inflammasome
Ubiquitination-related molecules	A20, PLK4, USP22, E3 ubiquitin ligases MARCH5, HECTD3, etc.	Regulate ubiquitination/deubiquitination of NEK7 or NLRP3, influencing inflammasome assembly
Other enzymes	Sirt5, OGT, METTL3	Affect the stability and activity of NEK7 or NLRP3 via acetylation, m6A modification, etc.
Ion channels and related proteins	P2X7R, Cl^-^ channels, K^+^ channels, etc.	Mediate ion efflux to promote NEK7-NLRP3 binding and trigger inflammasome activation
Small RNA–related	miR-340, miR-214-3p, miR-23a-3p, miR-181a-5p, etc.	Target NEK7 or its mRNA to suppress inflammasome activation
Other proteins	PAF, Aβ, S100A9, Foxo1, SLC25A3, cytochrome c, CGRP, RIPK1-IκBα, Epac1	Various mechanisms: promote or inhibit NEK7-NLRP3 binding and regulate signaling pathways
Bacterial/viral regulation	Pasteurella multocida, Staphylococcus aureus, Streptococcus suis, influenza A virus PB1-F2, SARS-CoV-2 ORF3a, rabies virus M protein	Partly promote (e.g., ion efflux) or partly inhibit (e.g., competitive binding to NLRP3 to suppress activation)

## 4 Targeted therapeutics for the NEK7-NLRP3 inflammasome

### 4.1 Natural Products and Derivatives Regulating the NEK7-NLRP3 Axis

#### 4.1.1 Quinone compounds

Quinone compounds have exhibited potent regulatory effects on NLRP3 inflammasome activation. Pristimerin, a quinone-type triterpenoid extracted from plants of the Celastraceae and Combretaceae families, inhibits inflammasome assembly by disrupting the interaction between NEK7 and NLRP3. Additionally, its α,β-unsaturated carbonyl moiety contributes to inflammasome deactivation (70). Hypocrellin A, a quinonoid sesquiterpene derived from Hypocrella bambusea, suppresses inflammasome activation via a dual mechanism: it inhibits ASC oligomerization and blocks direct NEK7-NLRP3 interaction by binding to key residues (Arg578 and Glu629) in the NACHT domain of NLRP3. Furthermore, it directly inhibits NLRP3 activation and has shown therapeutic potential in monosodium urate (MSU)-induced peritonitis and gouty arthritis models (71).

#### 4.1.2 Diterpenoid compounds

Diterpenoids represent a major class of natural products involved in the regulation of NLRP3 inflammasome activity. Oridonin, an active diterpenoid component isolated from Rabdosia rubescens, covalently binds to cysteine residues Cys277 and Cys279 of NLRP3, thereby blocking NEK7 interaction and suppressing inflammasome activation, as well as associated pathological processes such as neuroinflammation and pulmonary injury. A synthetic derivative, Compound 32, further inhibits ASC oligomerization and disrupts NEK7-NLRP3-ASC interactions, thereby blocking inflammasome assembly (72–76). Triptolide epoxide (triptolide diol), a diterpenoid epoxide from Tripterygium wilfordii, binds to NLRP3 at Cys280 with submicromolar affinity, preventing NEK7 interaction and reducing K63-linked ubiquitination. This binding induces a “closed” inactive conformation of NLRP3, thereby inhibiting inflammasome assembly (77). Rosthornin B, a diterpenoid isolated from Rabdosia species, directly targets NLRP3 to prevent its interaction with NEK7 and suppresses inflammasome assembly and activation. It exhibits significant pharmacological effects in LPS-induced septic shock models and MSU-induced peritonitis (78). Deacetylgedunin, a Gedunin-type limonoid derivative from Toona sinensis, exhibits lower cytotoxicity than its parent compound and significantly inhibits the interaction between NLRP3 and ASC/NEK7, thereby blocking inflammasome activation (79). Carnosic acid, a polyphenolic diterpenoid from rosemary (Rosmarinus officinalis), disrupts the NEK7-NLRP3 interaction to inhibit inflammasome assembly and activation, alleviating systemic inflammation and MSU-induced peritonitis in LPS-challenged models (80).

#### 4.1.3 Flavonoids and their derivatives

Flavonoids regulate NLRP3 inflammasome activation through multiple molecular targets. Quercetin-4′-O-β-D-glucoside (QODG), a flavonol glycoside, suppresses NEK7 succinylation by upregulating SIRT5, thereby disrupting the NEK7-NLRP3 interaction and alleviating high glucose-induced podocyte pyroptosis and oxidative stress injury (81). Interestingly, its aglycone form, quercetin, exerts the opposite effect by upregulating NEK7 expression, activating the NLRP3 inflammasome, and inducing pyroptosis in colorectal cancer cells (82). Tilianin, a flavonoid extracted from Dracocephalum moldavica L., inhibits NEK7-NLRP3 interaction through π-alkyl and hydrogen bonding interference. It also suppresses the TLR4/NF-κB signaling pathway and inflammasome activation, thereby offering cardioprotective effects against myocardial I/R injury (83). Farrerol, a bioactive flavonoid component derived from Rhododendron species, possesses antimicrobial and anti-inflammatory properties. It inhibits NLRP3 inflammasome activation by disrupting the NEK7-NLRP3 interaction, and alleviates myocardial I/R injury (84). Sinensetin, a polymethoxylated flavone found in citrus fruits, downregulates NEK7 expression and inhibits the Txnip/NLRP3/Caspase-1/GSDMD signaling axis, thereby reducing inflammation and pyroptosis in acute lung injury models (85). Gallic acid, an active phenolic acid, suppresses inflammasome assembly by blocking NEK7-NLRP3 interaction and ASC oligomerization, thus mitigating symptoms of gouty arthritis (86).

#### 4.1.4 Sesquiterpene lactones

Sesquiterpene lactones regulate NLRP3 inflammasome activation through direct interaction or structural interference. Britannin, a sesquiterpene lactone isolated from Inula britannica, inhibits inflammasome assembly by blocking the NEK7-NLRP3 interaction in an ATPase-independent manner. It is predicted to bind specifically to the NACHT domain of NLRP3 at residues Arg335 and Gly271. Britannin has demonstrated therapeutic efficacy in MSU-induced gouty arthritis and LPS-induced acute lung injury models (87). Artemisinin, a well-known sesquiterpene lactone derived from Artemisia annua, suppresses NLRP3 inflammasome activation by inhibiting NEK7-NLRP3 interaction in response to MSU stimulation (88). Helenin, a sesquiterpene lactone extracted from Inula helenium, contains an α, β-unsaturated carbonyl group that interferes with the NEK7-NLRP3 binding interface. It inhibits inflammasome assembly and activation, thereby alleviating LPS-induced lethal sepsis, systemic inflammation, and MSU-induced peritonitis (89).

#### 4.1.5 Triterpenoid saponins

Triterpenoid saponins exert their regulatory effects on the NLRP3 inflammasome by targeting NEK7 or associated signaling pathways. Ginsenoside Rg3, one of the major active components of Panax ginseng, inhibits the NEK7-NLRP3 interaction, thereby suppressing the NLRP3-ASC interaction, ASC oligomerization, and speck formation. This compound has been shown to alleviate endotoxin-induced shock in mouse models (90). Anemoside B4, the main bioactive saponin derived from Pulsatilla chinensis, targets NEK7 by inhibiting the NF-κB signaling pathway. It blocks NEK7-NLRP3 interaction and inflammasome assembly, suppresses pyroptosis, and reduces MSU-induced acute gouty arthritis (91).

#### 4.1.6 Alkaloids

Alkaloid compounds suppress NLRP3 inflammasome activation through epigenetic regulation or direct molecular interaction. Berberrubine hydrochloride, an isoquinoline alkaloid extracted from Coptis chinensis, structurally similar to berberine, inhibits METTL3-mediated m6A methylation to enhance the anti-inflammatory function of TNFAIP3. This promotes TNFAIP3-mediated ubiquitination and degradation of NEK7, thereby suppressing NLRP3 inflammasome activation and alleviating periodontal inflammation. Berberine (BBR), a well-known bioactive compound derived from traditional Chinese medicine (TCM), specifically disrupts the NEK7-NLRP3 interaction by forming a hydrogen bond between its 2,3-methylenedioxy group and the R121 residue of NEK7, which is abolished by R121A mutation (82, 92). Piperlongumine, an amide alkaloid, inhibits both NLRP3 oligomerization and the interaction between NLRP3 and NEK7 (93).

#### 4.1.7 Others

Rosmarinic acid, a natural polyphenol, alleviates acetaminophen-induced hepatotoxicity by inhibiting activation of the NEK7-NLRP3 signaling pathway (94). Manoalide, a marine-derived sesterterpenoid with potent analgesic and anti-inflammatory properties, covalently binds to lysine 377 (Lys377) of NLRP3 to block NEK7 interaction, thereby attenuating experimental autoimmune EAE in mice (95). TWLPLPR, a walnut-derived peptide, enhances the expression of synaptic plasticity-related proteins and suppresses NEK7-NLRP3 inflammasome signaling, thus ameliorating neuroinflammation and cognitive dysfunction associated with synaptic impairment in type 2 diabetic mice (96). Extracts from Trichospira verticillata reduce NEK7-NLRP3 binding, inhibit ASC oligomerization and speck formation, suppress inflammasome activation, and relieve symptoms of neutrophilic asthma in mouse models (66).

### 4.2 TCM NEK7–NLRP3 axis

Several TCM formulas have been shown to regulate NLRP3 inflammasome activity. Qishen Granules, derived from the classical prescriptions Zhenwu Decoction and Simiao Yong’an Decoction, are composed of Astragalus membranaceus, Salvia miltiorrhiza, Lonicera japonica, Scrophularia ningpoensis, Aconitum carmichaelii, and Glycyrrhiza uralensis. This formula inhibits macrophage inflammasome activation through the P2X7R-NEK7-NLRP3 signaling pathway and has been shown to be effective in the treatment of acute myocardial ischemia (97). Kui Jie Tong Formula, consisting of Verbena officinalis, Euphorbia humifusa, Areca catechu, Angelica sinensis, and Aurantii Fructus Immaturus, alleviates symptoms of ulcerative colitis by suppressing the classical NEK7-NLRP3-mediated pyroptotic pathway and modulating gut microbiota composition (98).

### 4.3 Synthetic compounds targeting the NEK7-NLRP3 axis

In recent years, significant progress has been made in the development of synthetic compounds targeting the NEK7-NLRP3 inflammasome axis. These compounds exert their effects by directly disrupting the NEK7-NLRP3 interaction, modulating inflammasome assembly, or regulating related signaling pathways. They have demonstrated promising therapeutic potential in the treatment of metabolic inflammation, neuroinflammation, and autoimmune diseases.

#### 4.3.1 Covalent or structural inhibitors directly targeting the NEK7-NLRP3 interaction

This class of compounds directly disrupts the NEK7-NLRP3 interaction interface through covalent bonding or steric hindrance, representing one of the most direct strategies for modulating the inflammasome complex.

Rociletinib, a covalent EGFR inhibitor, has shown significant effects in modulating NLRP3-mediated metabolic inflammation in high-fat diet-induced type 2 diabetic mouse models. Mechanistically, ROC covalently binds to cysteine 79 (Cys79) of NEK7 via the electrophilic α,β-unsaturated carbonyl group within its N-phenylacrylamide moiety, thereby blocking the NEK7-NLRP3 interaction and inhibiting inflammasome assembly and activation (99).

Entrectinib, a tyrosine kinase inhibitor, directly binds to arginine 121 (R121) of NEK7, disrupting the NEK7-NLRP3 interaction interface and suppressing inflammasome assembly and activation. It has demonstrated anti-inflammatory effects in models of systemic inflammation, peritonitis, and type 2 diabetes (100).

Compound 149-01, a derivative of RRx-001 lacking high-energy nitro functional groups, covalently targets cysteine 409 (Cys409) of NLRP3 to block NEK7 binding, thereby preventing NLRP3 inflammasome activation (101).

MCC950, a classic NLRP3 inhibitor, binds to the LRR domain of NLRP3 and stabilizes it in a “closed” inactive conformation, which prevents NEK7 engagement and inflammasome activation (102, 103).

INF39, an irreversible acrylate-based inhibitor, specifically disrupts the NEK7-NLRP3 interaction and further inhibits downstream steps of inflammasome assembly, including NLRP3 oligomerization, NLRP3–ASC binding, and ASC speck formation (104).

1,2-Diol (4-phenyl-indole derivative) inhibits inflammasome assembly by disrupting the NEK7-NLRP3 interface and attenuates LPS-induced acute lung injury (105).

ODZ10117 and 10,11-Dehydrocurvularin both inhibit inflammasome activation by directly blocking the NEK7-NLRP3 interaction, although the precise binding sites and mechanisms remain under investigation (106, 107).

Virtual screening compounds I-19 and II-8, identified via high-throughput virtual screening, bind tightly to the LRR domain of NLRP3. Through steric hindrance, they block the NEK7-NLRP3 interaction and effectively suppress aberrant inflammasome activation in rheumatoid arthritis models (108).

#### 4.3.2 Indirect inhibitors regulating NEK7 expression or function

These compounds modulate the NEK7-NLRP3 inflammasome complex indirectly by altering NEK7 expression levels or interfering with its downstream signaling pathways.

Vortioxetine, a multimodal antidepressant, has been found to reduce LPS-induced expression of NLRP3 and ASC specifically in memory-associated brain regions such as the dorsal hippocampus. This regulatory effect is likely mediated by the downregulation of NEK7 expression, with no observed impact on other inflammasomes (109).

Metformin, a classical anti-diabetic drug, reduces NLRP3 inflammasome activity by suppressing NEK7 expression. This reduction may be linked to metformin-induced cell cycle arrest. Additionally, in diabetic periodontitis models, metformin alleviates NLRP3-mediated pyroptosis by downregulating NEK7 expression (110).

Melatonin exerts anti-inflammatory effects by modulating the transcriptional activation of pro-inflammatory mediators. Its mechanism likely involves the inhibition of NEK7-NLRP3 and TLR2 expression, thereby reducing lung tissue damage (111).

#### 4.3.3 Multi-target compounds inhibiting inflammasome assembly

Several multi-target drugs have been shown to inhibit NLRP3 inflammasome assembly by interfering with ASC oligomerization, NLRP3 oligomerization, or NEK7-NLRP3 interactions.

Leukadherin-1, an anti-cancer compound, suppresses NLRP3 inflammasome assembly by inhibiting ASC oligomerization, blocking NLRP3 complex formation, and reducing NEK7-NLRP3 interactions (112).

SLC3037 inhibits inflammasome activation triggered by MSU crystals and other stimuli. Its mechanism involves blocking NEK7-NLRP3 binding or NLRP3 oligomerization, thereby interfering with subsequent ASC oligomerization and phosphorylation processes (113).

SB-222200 binds directly to the NLRP3 protein, preventing NEK7-NLRP3 interaction and NLRP3 oligomerization. It has been shown to alleviate MSU-induced peritonitis and dextran sulfate sodium-induced acute intestinal inflammation (114).

Candesartan, an angiotensin II receptor antagonist, reduces mitochondrial damage and inhibits NLRP3 inflammasome assembly by blocking the binding of NLRP3 to PKR, NEK7, and ASC (115).

Dimethyl fumarate, a first-line drug for relapsing-remitting multiple sclerosis, covalently modifies the Cys673 residue of NLRP3, thereby inhibiting its interaction with NEK7 and blocking inflammasome activation induced by various stimuli (116).

LCC18, a benzamide-linked small molecule, prevents ASC oligomerization and NLRP3 inflammasome assembly by inhibiting the binding of NLRP3 to PKR, NEK7, and ASC, and has shown therapeutic effects in mouse models of IgA nephropathy (117).

Ascorbyl Palmitate, a lipophilic derivative of ascorbic acid, acts as an antioxidant that directly scavenges mtROS. It also blocks the NEK7-NLRP3 interaction and inflammasome assembly, thereby alleviating inflammatory disease progression (118).

#### 4.3.4 Indirect inhibitors modulating the redox state of NEK7

C1-27, a small-molecule inhibitor of glutathione transferase omega 1-1 (GSTO1-1), suppresses NLRP3 inflammasome activation by modulating the redox state of NEK7. GSTO1–1 acts as a deglutathionylase that targets cysteine 253 (Cys253) of NEK7, promoting inflammasome activation. In contrast, C1–27 inhibits GSTO1–1 activity, thereby preventing NEK7 deglutathionylation and indirectly suppressing NLRP3 inflammasome activation (119).

N-Acetyl-L-cysteine, a well-known ROS scavenger, markedly inhibits NEK7-NLRP3 inflammasome activation in response to uric acid stimulation and alleviates apoptosis in renal tubular epithelial cells (120). In summary, synthetic compounds targeting the NEK7-NLRP3 inflammasome complex have evolved into a multi-dimensional therapeutic strategy. These include direct disruption of NEK7-NLRP3 interactions, stabilization of NLRP3 inactive conformations, transcriptional regulation of NEK7 expression, and modulation of NEK7 redox state. Collectively, these compounds offer a diverse repertoire of therapeutic candidates for treating metabolic inflammation, neurodegenerative diseases, and autoimmune disorders.

### 4.4 Biologics and other novel tools

TRIM65, an immunoregulatory protein, binds to the NACHT domain of NLRP3 and promotes its lysine 48- and lysine 63-linked ubiquitination. This modification inhibits the interaction between NEK7 and NLRP3, thereby suppressing NLRP3 inflammasome assembly (32).

4-Hydroxy-2-nonenal, a major endogenous product of lipid peroxidation, directly binds to NLRP3 and inhibits its interaction with NEK7, suppressing pyroptosis and NLRP3 inflammasome activation (121).

Pickering emulsions have been shown to bind to NEK7 or NLRP3, interfering with NLRP3 inflammasome assembly and thereby alleviating inflammation (122).

In addition, 30-nanometer gold nanoparticles can selectively disrupt NLRP3 inflammasomes in bone marrow-derived macrophages without affecting other inflammasomes. This effect may be mediated by the clearance of ROS, which blocks the NEK7-NLRP3 interaction (123).

As summarized in **Table 2**, a variety of traditional Chinese medicines, natural products and their derivatives, synthetic compounds, biologics, and novel nanomaterials have been developed to target different mechanisms involved in NEK7-mediated NLRP3 inflammasome activation.

**Table 2:** Drugs targeting NEK7-NLRP3 inflammasome activation.

Drug category	Representative drugs/compounds	Mechanistic description
Traditional Chinese Medicines and Natural Products	Oridonin, Britanin, Panax notoginseng saponins, Hypocrellin A, Tripterygium diterpenes, Magnolol, Quercetin-4′-O-β-D-glucoside, Ginkgolic acid B, Tilianin, Farrerol, Gallic acid, Deacetylgedunin, Sinensetin, Rosthornin B, Manoalide, Piperlongumine, TWLPLPR, Carnosic acid; Zhenwu Decoction, Simiao Yong’an Decoction, Kui Jie Tong Formula, etc.	Inhibit NLRP3 inflammasome assembly by blocking NEK7-NLRP3 interactions, or modulate activation to exert anti-inflammatory effects
Synthetic Compounds	Rociletinib, Vortioxetine, Entrectinib, Leukadherin-1, MCC950, SLC3037, SB-222200, Candesartan, Dimethyl fumarate, INF39, 1,2-Diol, ODZ10117, 10,11-Dehydrocurvularin, C1-27, NAC, Compounds omega1-1, 149-01, I-19, II-8, LCC18, etc.	Bind to specific NEK7 sites or regulate its expression, thereby suppressing NLRP3 inflammasome activation
Biologics and Others	TRIM65, 4-hydroxy-2-nonenal, anti-VEGF aflibercept, Pickering emulsions, gold nanoparticles, etc.	Inhibit NEK7-NLRP3 interactions through various mechanisms, such as regulating ubiquitination, oxidative state, or competitive binding

## 5 Conclusion and perspectives

This review summarizes current advances in understanding the molecular mechanisms, regulatory networks, and therapeutic strategies associated with NEK7-mediated activation of the NLRP3 inflammasome. Increasing evidence indicates that the interaction between NEK7 and NLRP3 functions as a critical molecular licensing step for inflammasome assembly. Structural studies have revealed that the catalytic domain of NEK7 binds to the LRR domain of NLRP3, triggering conformational rearrangement that exposes the PYD domain and enables ASC recruitment and caspase-1 activation. This conformational activation represents a fundamental mechanism governing NLRP3 inflammasome assembly and provides an essential structural basis for inflammatory signaling.

Despite significant progress, several key aspects of NEK7-NLRP3 conformational activation remain incompletely understood. For example, the precise structural dynamics that control the transition of NLRP3 from an autoinhibited to an active conformation are still unclear. In addition, how diverse upstream signals—including ionic flux, post-translational modifications, metabolic stress, and pathogen-derived stimuli—are integrated to regulate NEK7-dependent conformational changes remains to be fully elucidated. The spatiotemporal organization of the NEK7-NLRP3 complex within cellular compartments, such as mitochondria and the microtubule-organizing center (MTOC), also remains to be fully elucidated.

Form a therapeutic perspective, the NEK7-NLRP3 axis represents an attractive target for anti-inflammatory drug development. Advances in structural biology, particularly cryo-electron microscopy, have facilitated the identification of key interaction interfaces within the NEK7–NLRP3 complex and enabled the development of several small-molecule inhibitors (124). Representative examples include natural products such as oridonin, synthetic inhibitors such as MCC950 that stabilize the inactive conformation of NLRP3, as well as emerging biologics targeting upstream inflammatory signaling pathways. Future drug development may benefit from disease-context–specific strategies, as distinct pathological conditions may involve different inflammasome activation mechanisms. For instance, metabolic disorders are often associated with mitochondrial stress and ROS-dependent activation, whereas neurodegenerative diseases are frequently linked to chronic microglial activation and lysosomal dysfunction. These differences suggest that next-generation inhibitors may selectively target specific activation nodes within the NEK7–NLRP3 signaling cascade, such as the NEK7–NLRP3 interaction interface, NACHT ATPase activity, or upstream stress-sensing pathways.

However, the development of clinically effective NEK7–NLRP3 inhibitors remains challenging. One major difficulty lies in targeting the protein–protein interaction interface between NEK7 and NLRP3, which involves large and dynamic binding surfaces. In addition, the structural plasticity of NLRP3 and the context-dependent activation of the inflammasome complicate the design of highly selective inhibitors. Pharmacokinetic limitations, particularly insufficient blood–brain barrier (BBB) penetration, also represent significant barriers for treating neuroinflammatory disorders. Potential strategies to overcome this challenge include optimization of small-molecule physicochemical properties, nanoparticle-based drug delivery systems, and receptor-mediated transport approaches to improve CNS drug distribution.

Future studies integrating structural biology, chemical biology, and systems immunology will be essential to further elucidate the conformational activation mechanism of the NEK7–NLRP3 complex. Emerging technologies—including *in situ* cryo-electron microscopy, molecular dynamics simulations, and AI-assisted drug discovery—may help resolve the dynamic structural landscape of inflammasome activation and accelerate the development of next-generation therapeutic agents targeting the NEK7–NLRP3 axis.

## Glossary

ALK: Anaplastic lymphoma kinase
MARCH5: Membrane-associated RING-CH-type finger 5
ASC: Apoptosis-associated speck-like protein containing a CARD
MCC950: a classic NLRP3 inhibitor
ASK1: Apoptosis signal-regulating kinase 1
METTL3: Methyltransferase-like 3
ATP: Adenosine triphosphate
MSU: monosodium urate
BBB: Blood–brain barrier
Mst 1/2: mammalian sterile 20-like kinases 1 and 2
BBR: Berberine
MTOC: Microtubule-organizing center
CAPS: Cryopyrin-associated periodic syndrome
NACHT: Nucleotide-binding and oligomerization domain
CARD: Caspase activation and recruitment domain
NEK7: NIMA-related kinase 7
CASP8: Caspsse 8
NLRP3: NOD-like receptor family pyrin domain containing 3
CGRP: Calcitonin gene-related peptide
OGT: O-linked N-acetylglucosamine transferase
DAMPs: damage-associated molecular patterns
PAMPs: pathogen-associated molecular patterns
EAE: Experimental autoimmune encephalomyelitis
PAF: Platelet-activating factor
EGFR: Epidermal growth factor receptor
PKA: Protein kinase A
GBP5: Guanylate-binding protein 5
PKR: Double-stranded RNA-dependent protein kinase
GSDMD: Gasdermin D
QODG: Quercetin-4′-O-β-D-glucoside
GSTO1-1: glutathione transferase omega 1-1
RACK1: Receptor for activated C kinase 1
Hel: Helenin
RIPK1: Receptor-interacting serine/threonine-protein kinase 1
HECTD3: HECT domain E3 ubiquitin ligase 3
Ros B: Rosthornin B
HMGB1: High mobility group box 1
ROS: Reactive oxygen species
IAV: Influenza A virus
TCM: Traditional Chinese Medicine
IL: Interleukin
TLR4: Toll-like receptor 4
INF39: an irreversible acrylate-based inhibitor
TNF: Tumor necrosis factor
LATS1/2: large tumor suppressor kinases 1 and 2
TNFAIP3: Tumor necrosis factor alpha-induced protein 3
LPS: Lipopolysaccharide
TRIM65: Tripartite motif-containing protein 65
LRR: Leucine-rich repeat
USP15: Ubiquitin-specific peptidase 15
PLK4: Polo-like kinase 4
USP22: Ubiquitin-specific peptidase 22
PTMs: post-translational modifications
ZDHHC17: Palmitoyltransferase ZDHHC17
PYD: Pyrin domain
